# Reassessment of Iron Biomarkers for Prediction of Dialysis Iron Overload: An MRI Study

**DOI:** 10.1371/journal.pone.0132006

**Published:** 2015-07-16

**Authors:** Guy Rostoker, Mireille Griuncelli, Christelle Loridon, Théophile Magna, Gabrielle Machado, Gilles Drahi, Hervé Dahan, Philippe Janklewicz, Yves Cohen

**Affiliations:** 1 Division of Nephrology and Dialysis, Ramsay Générale de Santé, Hôpital Privé Claude Galien, Quincy sous Sénart, France; 2 Department of Biochemistry, Ramsay Générale de Santé, Hôpital Privé Claude Galien, Quincy sous Sénart, France; 3 Division of Radiology, Ramsay Générale de Santé, Hôpital Privé Claude Galien, Quincy sous Sénart, France; Sao Paulo State University, BRAZIL

## Abstract

**Background and Objectives:**

Iron overload among hemodialysis patients was previously considered rare but is now an increasingly recognized clinical situation. We analyzed correlations between iron biomarkers and the liver iron concentration (LIC) measured by magnetic resonance imaging (MRI), and examined their diagnostic accuracy for iron overload.

**Design, Setting, Participants and Measurements:**

We performed a prospective cross-sectional study from 31 January 2005 to 31 August 2013 in the dialysis centre of a French community-based private hospital. A cohort of 212 hemodialysis patients free of overt inflammation or malnutrition, were treated for anemia with parenteral iron-sucrose and an erythropoesis-stimulating agent, in keeping with current clinical guidelines. Blinded measurements of hepatic iron stores were performed by T1 and T2* contrast MRI, and relationships were analysed using Spearman’s coefficient, logistic regression and receiver-operator characteristic (ROC) curves.

**Results:**

Among the biological markers, only serum ferritin showed a strong correlation with LIC (rho= 0.52, 95% CI: 0.41-0.61, p< 0.0001, Spearman test). In logistic analysis, only serum ferritin correctly classified the overall cohort into patients with normal liver iron stores (LIC ≤ 50 μmol/g) and those with elevated liver iron stores (LIC > 50 μmol/g) (odds ratio 1.007; 95% CI: 1.004-1.010). Serum ferritin was the iron biomarker with the best discriminatory capacity in ROC curves analysis (area under the curve (AUC) = 0.767; 95% CI: 0.698-0.835). The optimal serum ferritin cutoffs were 160 μg/L for LIC > 50 μmol/g (mild iron overload) and 290 μg/L for LIC > 200 μmol/g (severe iron overload).

**Conclusions:**

For clinical purposes, serum ferritin correctly reflects liver iron stores, as assessed by MRI, in hemodialysis patients without overt inflammation or malnutrition. These results strongly suggest that current ferritin target values should be lowered to avoid iron overload.

**Trial Registration:**

ISRCTN Registry 80100088

## Introduction

Routine use of recombinant erythropoiesis-stimulating agents (ESA) has enabled anemia to be corrected in dialysis patients during the past two decades, thereby improving their quality of life and permitting better outcomes [1]. As successful use of ESA requires sufficient available iron, almost all end-stage renal disease patients on ESA receive concomitant parenteral iron therapy [1,2]. Iron overload among dialysis patients in the ESA era was previously considered rare [1–3]. We recently challenged this view, showing that 84% of 119 unselected hemodialysis patients had hemosiderosis, based on quantitative magnetic resonance imaging (MRI) of liver iron stores, and that 30% of them had severe iron overload at levels seen in genetic hemochromatosis [4].

The only laboratory parameter available to screen for iron overload in dialysis patients is serum ferritin, but its validation requires liver or bone marrow biopsy, and few data are available for patients with end-stage renal disease because of the associated risks or aggressiveness of these invasive procedures [5]. Moreover, serum ferritin is an acute-phase reactant, and these patients’ frequent systemic inflammation can markedly interfere with its measurement and also inhibit both iron mobilization from reticuloendothelial stores and intestinal iron absorption via hepcidin modulation [5]. The increasing prevalence of multiple comorbidities in the population of dialysis patients has also made the use of serum ferritin as a biomaker more challenging in recent years [6]. Finally, comparisons of potential biological markers of excess iron stores with gold-standard methods must also take into account the paradoxical fact that, in hemodialysis patients receiving intravenous iron, bone marrow iron content may be low despite severe hepatosplenic siderosis in up to a one-third of cases [3]. Thus, liver iron content seems to be the best indicator of iron overload in hemodialysis patients, while bone marrow analysis may be misleading [3]. The liver is the main site of iron storage in humans, and the liver iron concentration (LIC) correlates closely with total body iron stores in healthy persons as well as patients with genetic hemochromatosis and secondary hemosideroses such as thalassemia major and sickle cell disease [7]. It seems very likely that iron overload in hemodialysis patients follows the same rules [3,4,8,9]. In systemic iron overload, up to 70% to 90% of total body iron stores are found primarily in hepatocytes and Kupffer cells, mainly as ferritin and hemosiderin iron [7,9]. Hepatic MRI has now emerged as the gold-standard method for estimating and monitoring iron stores in genetic hemochromatosis and secondary hemosideroses [9]. Moreover, epidemiological studies have recently shown that excessive parenteral iron administration can also adversely affect the prognosis of hemodialysis patients [10–12].

Iron overload in hemodialysis patients may be favored by reimbursement policies in the USA and many other developed countries that have led to a dramatic increase in the use of intravenous iron preparations in order to avoid the high costs of ESA therapy; this situation may also be aggravated by excessive advocated doses of intravenous iron and erroneous iron biomarker targets aimed at “repleting” iron stores [4,8,13]. Indeed, we have recently shown that the standard maximal amount of iron infused per month should be lowered to 250 mg in order to lessen the risk of dialysis iron overload and to allow safer use of parenteral iron products [13].

The aim of this present study was therefore to determine, in our original cross-sectional cohort of 119 hemodialysis patients treated with ESA and parenteral iron and studied by hepatic MRI, and in 94 new patients studied in the same way, the reliability of biological markers to detect iron overload, by comparison with LIC measured by MRI [4,13].

## Materials and Methods

### Patients and dialysis

**Fig 1:**
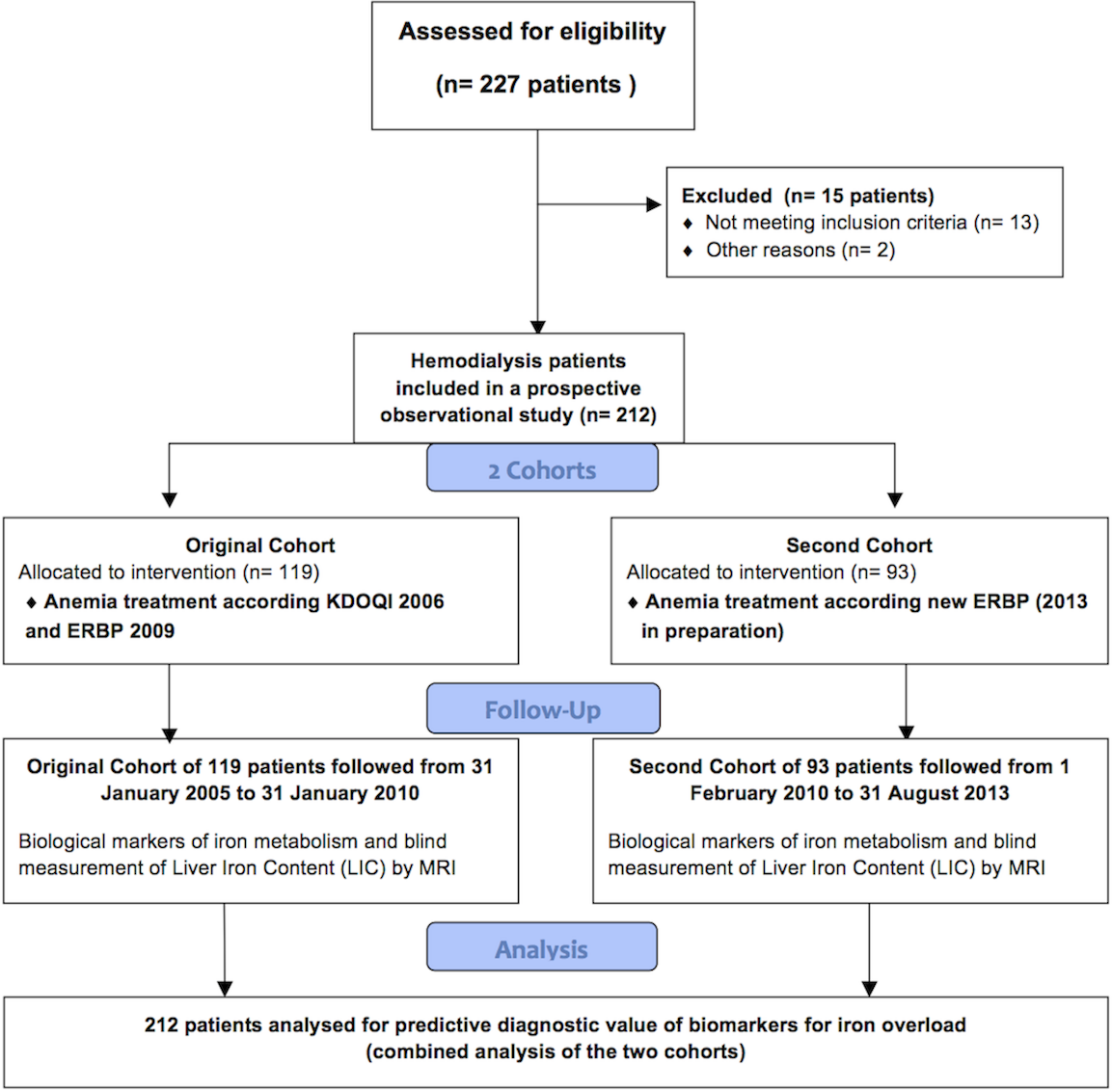
Consort flow Diagram.

With the patients’ informed consent and ethical approval from the Drug, Devices and Clinical Trials Committee of our institution (COMEDIMS Claude Galien, 9 December 2004), 119 fit patients free of overt inflammation or malnutrition and undergoing chronic intermittent bipuncture bicarbonate hemodialysis three times a week (at Claude Galien’s dialysis unit) with ultrapure dialysate and single-use biocompatible membranes were enrolled in this prospective cross-sectional study during a 60-month period, from 31 January 2005 to 31 January 2010. The exclusion criteria were as follows: refusal to participate in the study, poor compliance with the dialysis therapy schedule, age <18 years, severe cognitive impairment, claustrophobia, hepatic cirrhosis, overt inflammation (C-reactive protein > 125 mg/L) or infectious disease, malnutrition (albuminemia < 30 g/L), recent major bleeding (< 3 months), major surgery (< 3 months), transfusion dependency, recent transfusion (< 3 months), intractable malignancy, cardiac pacemakers and metallic cardiac valves. The participants provided their written consent after having received verbal explanations from their nephrologist, together with a detailed information sheet. Signed informed consent forms were kept in a loose-leaf file. These procedures were approved by the Drug, Devices and Clinical Trials Committee of our institution. These 119 patients were the subject of a recent publication highlighting the risk of iron overload in this setting; the methodology of this cross-sectional study is described in depth in our published article [4]. It is of note that 14 patients screened for participation in the original study were excluded, 12 for technical contraindications to MRI (10 with a cardiac pacemaker; 1 for metallic debris in the eyes; 1 for claustrophobia), and 2 for repeated non attendance at MRI appointments (Fig 1).

For the present study a second cohort comprising a further 94 fit hemodialysis patients studied in the same way was composed from 1 February 2010 to 31 August 2013. One patient in the second cohort was excluded from the analysis because he had a pelvic abscess accompanied by a major increase in inflammatory markers at the time of hepatic MRI. The second cohort therefore comprised 93 patients (Fig 1).

In keeping with the European Best Practice guideline, from 31 January 2005 to 31 January 2010, anemia treatment in our hemodialysis centre comprised once-weekly intravenous administration of darbepoetin alpha and, if required, 100 mg of iron-sucrose (Venofer vial, 100 mg/5 ml Vifor International, Villars sur Glâne, Switzerland) starting twice to three times a week (induction phase), then once a week to once every four weeks (maintenance phase), with the following targets: hemoglobin 10–12 g/dL; transferrin saturation (TSAT): lower limit: 20%, target range 30%-50%; and serum ferritin: lower limit: 100 μg/L, target range: 200 to 500 μg/L [2]. For the patients enrolled in the second cohort (patient # 120–212), the results of our published study on the risk of iron overload and our call for a revision of guidelines in this area led us to anticipate the new European guideline which set upper targets for ferritin at 300 μg/L and TSAT at 30%, and a target hemoglobin of 10–12 g/dL, and did not fully endorse the KDIGO guideline [4,6,14]. Moreover, for economic reasons, iron-sucrose Venofer was replaced in our hospital by a generic (iron-sucrose-Actavis), as authorised by the European Medicines Agency (Fig 1). Therefore, the second cohort (patients #120–212) received the generic while the 119 patients in the original cohort received Venofer. It is of note that we recently demonstrated a similar risk of liver accumulation with these two iron-sucrose pharmaceuticals (eg the original Venofer and its generic iron-sucrose—Actavis) [13] (Fig 1).

This study was registered under International Standard Randomized Controlled Trial Number (ISRCTN, reference 80100088).

We did not register this study before the first enrolments began, as registration of observational studies was rarely done at that time and observational studies were generally not considered as clinical trials. The authors solemnly declare that all ongoing and related trials for this drug/intervention are registered.

### Quantitative magnetic resonance imaging of hepatic iron stores

We used a signal intensity ratio method based on T1 and T2* contrast imaging without gadolinium, as established by Gandon and coworkers at Rennes University [15]. Patients on iron therapy received their iron dose at least one week before MRI. MRI measurements were made by four senior radiologists (PJ, YC, HD and GD) who were unaware of the patients’ medical history and biochemical results. The upper 95% of LIC in healthy adults is 32 μmol/g of dry liver, but as hepatic MRI accurately detects liver iron overload exceeding 50 μmol/g of dry liver, the upper limit of normal was set at 50 μmol/g for this study [4,15]. LIC values between 51 and 100 μmol/g were considered to represent mild iron overload, LIC values between 101 and 200 μmol/g moderate iron overload; and LIC values > 200 μmol/g of dry liver severe iron overload [4,15]. These LIC cutoffs are evidence-based with respect to previous and current data for liver biopsy and MRI; these gradual categories of iron overload reflect an increasing risk of complications in iron-overload disorders such as genetic hemochromatosis and secondary hemosiderosis related to hematological disorders [9].

### Biological markers of iron metabolism

Anemia treatment efficacy was estimated by hemoglobin assay and reticulocyte counts every two weeks, as well as monthly measurements of iron biomarkers (ferritin, transferrin, serum iron and transferrin saturation (TSAT)) and C-reactive protein. Soluble transferrin receptors (sTfR) were measured monthly in the original cohort and quarterly in the second cohort. Serum hepcidin-25 was measured once in the two cohorts, at the outset of a mid-week dialysis session immediately following MRI. All the blood samples for measurement of biological markers of iron metabolism were obtained at the outset of the mid-week dialysis session, at least seven days after the last iron infusion. As advocated by Skikne, we also calculated the ratio of sTfR to ferritin, which is only marginally affected by inflammation [16]. The assay methods and normal ranges of these biological markers are given in the table of results. Finally, we also analyzed erythrocyte mean corpuscular volume, which was shown by Gokal and co-workers at the end of the 1970s to be informative of dialysis post-transfusional iron overload [17]. With the exception of hepcidin-25 (in both the original and second cohorts), soluble transferrin receptors and the sTfR/ferritin ratio (only in the second cohort), statistical analyses used the median of three values for each biomarker, obtained the same month as hepatic MRI and one month before and one month after MRI.

### Statistical analyses

As values did not conform to a Gaussian distribution (Shapiro-Wilk normality test), all data are expressed as medians and ranges; percentages are given with their 95% confidence intervals calculated with the modified Wald method [18]. The two cohorts of patients were compared by using the non-parametric two-tailed Mann and Whitney test for continuous variables and the chi-square test for categorical variables [18].

Univariate correlations among the biological markers of iron metabolism and MRI LIC were studied with Spearman’s rank-order correlation coefficient [18].

Prism 6 software (Graphpad, San Diego, USA) was used for all tests, and p values < 0.05 were considered to denote statistical significance [18].

As an exploratory tool, we analyzed the capacity of the iron biomarkers to correctly classify hemodialysis patients as having normal (≤ 50 μmol/g dry weight) or elevated (> 50 μmol/g dry weight) liver iron content on MRI, by using stepwise backward Wald binary logistic regression analysis (SPSS software from IBM Bois-Colombes, France) in the whole cohort of 212 patients, with the aim of increasing the power of the study and of reducing the risk of spurious results related to small sample sizes [18]. We also performed binary logistic regression analyses with and without outliers [18].

Finally, we used receiver-operator characteristic (ROC) curves to analyze the capacity of the biological markers of iron metabolism to predict MRI-based hepatic iron overload, and to identify optimal test and threshold values (XLSTAT-Life from Addinsoft, Paris, France), in the whole cohort of 212 patients, again with the aim of increasing the power of the study and of reducing the risk of spurious results related to small sample sizes [19]. The optimal threshold value of each iron biomarker was determined, as recently advocated, by calculating the highest modified Younden’s index, taking into account the sum of specificity and sensitivity [20].

To analyze the potential benefit of combinations of iron biomarkers for diagnosis of iron overload on MRI, we established a ROC curve combining seven iron markers (serum iron, ferritin, transferrin, TSAT, serum soluble transferrin receptor, STfR/ferritin ratio, and hepcidin) and C-reactive protein, using predicted probabilities derived from the binary logistic regression [18].

## Results

### Characteristics of the study population

**Fig 2:**
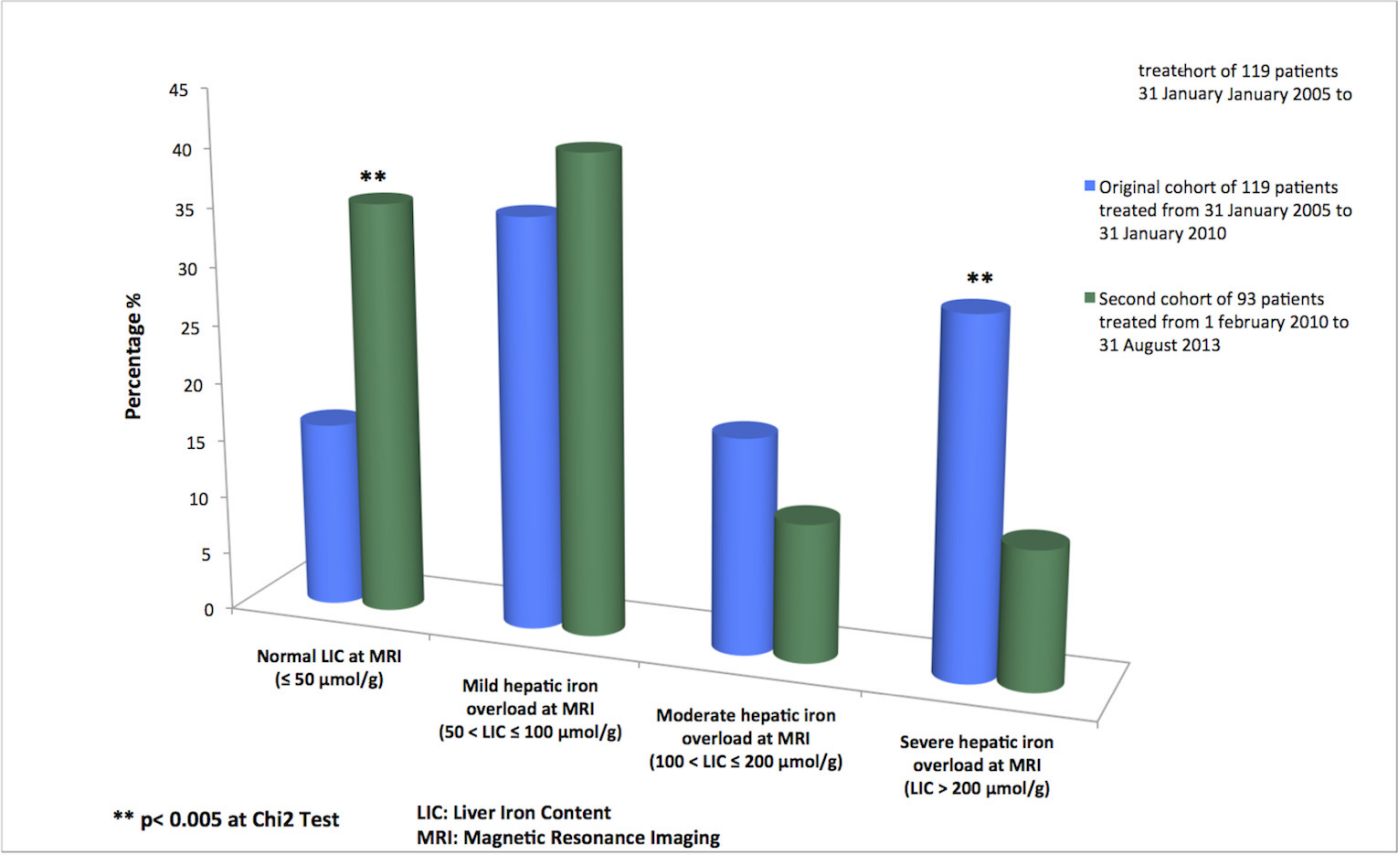
Histogram of liver iron content measured by quantitative MRI in two cohorts of hemodialysis patients treated with different ferritin and transferrin saturation targets for iron repletion.

Baseline characteristics, clinical findings and biological data for the two patient cohorts are summarized in Tables 1 and 2 and in Fig 2. These data are described and discussed in depth in our recently published article (in PLoS One) [13]. No adverse effects of MRI occured; moreover, patients’ knowledge of their LIC did not have deleterious psychological effects.

**Table 1:** Characteristics and findings in 212 hemodialysis patients studied by hepatic MRI to determine liver iron content (LIC), and assessment of iron biomarkers.

Variables	Overall Cohort (n°1 + n°2)(n = 212)	Cohort n°1 (n = 119)	Cohort n°2 (n = 93)	P value at Mann and Whitney test or Chi2test at comparison of cohort 1 and 2
age (years)	64 [19–91]	60 [19–87]	69 [20–91]	p = 0.0014 at Mann and Whitney test
Female sex, percentage of patients (%)	37.26 [31.03–43.95]	38.66 [30.38–47.64]	35.48 [26.50–45.62]	p = 0.7408 at X2 test
Dialysis vintage (months)	11.50 [1–95]	16 [2–95]	10 [1–86]	p = 0.0031 at Mann and Whitney test
ESA Therapy, percentage of patients (%)	97.64 [94.44–99.14]	99.16 [94.93–99.99]	95.70 [89.11–98.66]	p = 0.2334 at X2 test
Darbepoetin Dose (microg/month)	143 [0–775]	130 [0–566]	157.80 [0–775]	p = 0.0085 at Mann and Whitney test
Parenteral iron therapy, percentage of patients (%)	91.51 [86.91–94.63]	94.96 [89.21–97.90]	87.10 [78.64–92.61]	p = 0.0735 at X2 test
Iron dose (mg/month)	225 [0–900]	169.20 [0–900]	303.20 [0–790]	p< 0.0001 at Mann and Whitney test
Liu Comorbidity index	3 [0–13]	3 [0–13]	3 [0–11]	p = 0.6804 at Mann and Whitney test
Charlson Comorbidity index	6 [2–16]	6 [2–16]	7 [2–16]	p = 0.0213 at Mann and Whitney test
Diabetes, percentage of patients (%)	28.77 [23.09–35.21]	22.69 [16.04–31.05]	36.56 [27.47–46.71]	p = 0.0393 at X2 test
Audit Score	2 [0–40]	2 [0–40]	2 [0–40]	p = 0.3347 at Mann and Whitney test
Normal LIC at MRI, percentage of patients (%) (LIC ≤ 50 micromol/g at MRI)	24.53 [19.21–30.76]	15.97 [10.38–23.68]	35.48 [26.50–45.62]	p = 0.0018 at X2 test
Mild hepatic iron overload at MRI, percentage of patients (%) (LIC: 51 to 100 micromol/g at MRI)	37.74 [31.48–44.43]	35.29 [27.28–44.23]	40.86 [31.42–51.03]	p = 0.4921 at X2 test
Moderate hepatic iron overload at MRI, percentage of patients (%) (LIC: 101 to 200 micromol/g at MRI)	15.57 [11.27–21.09]	18.49 [12.47–26.48]	11.83 [6.57–20.11]	p = 0.2558 at X2 test
Severe hepatic iron overload at MRI, percentage of patients (%) (LIC > 200 micromol/g at MRI)	22.17 [17.08–28.25]	30.25 [22.70–39.04]	11.83 [6.57–20.11]	p = 0.0024 at X2 test

MRI: Magnetic Resonance Imaging.

LIC: Liver Iron Content.

Values are given as median and [range].

**Table 2:** Biochemical markers of iron metabolism in 212 hemodialysis patients studied by hepatic MRI to determine liver iron content (LIC).

Variables	Overall Cohort (n°1 + n°2)(n = 212)	Cohort n°1 (n = 119)	Cohort n°2 (n = 93)	P Value at Mann and Whitney test or Chi2 test at comparison of cohort 1 and 2
Hemoglobin (g/dL), Advia 2120, Siemens, Normal range in dialysis patients: [10–12 g/dL]	11.53 [8.38–15.12]	11.97 [8.43–15.12]	11.08 [8.38–14.68]	p = 0.0012 at Mann and Whitney test
Erythrocyte mean corpuscular volume (fL), Advia 2120, Siemens, Normal range: [82–98 fL]	94 [66–118.20]	94 [66–108]	94.33 [76.50–118.20]	p = 0.7477 at Mann and Whitney test
C-reactive protein (mg/L), Immunoturbidimetry using latex particles, Roche Diagnostics; Normal range: [< 5 mg/L]	4.05 [0.30–107.30]	4.30 [0.30–75.93]	3.90 [1–107.30]	p = 0.7545 at Mann and Whitney test
Serum ferritin (microg/L), Immunoturbidimetry using latex particles, Roche Diagnostics; Normal range: [M: 30–400 microg/L, F: 15–150 microg/L]	206.20 [12–2229]	265.50 [15–1383]	149 [12–2229]	p = 0.0020 at Mann and Whitney test
Serum iron (micromol/L), Colorimetric test, Roche Diagnostics; Normal range: [M: 11–28, F: 6.6–26 micromol/L]	10.12 [3.59–26.27]	9.65 [3.59–26.27]	10.57 [4.18–26.27]	p = 0.2832 at Mann and Whitney test
Serum transferrin (g/L), Immunoturbidimetry, Roche Diagnostics; Normal range: [2–3.6 g/L]	1.83 [1.07–4.47]	1.69 [1.07–2.77]	1.95 [1.23–4.47]	p< 0.0001 at Mann and Whitney test
Transferrin saturation (TSAT)(%), serum iron/Total iron-binding capacity ratio; Normal range: [M: 20–40%, F: 15–35%]	22.67 [6.33–72.16]	23.07 [6.33–72.16]	21.75 [6.50–61.17]	p = 0.1746 at Mann and Whitney test
Serum soluble transferrin receptor (sTfR)(mg/L) Immunoturbidimetry using latex particles, Roche Diagnostics; Normal range: [M: 2.2–5, F: 1.9–4.4 mg/L]	4.84 [0.48–13.02]	4.27 [1.43–13.02]	5.38 [0.48–12.84]	p = 0.0114 at Mann and Whitney test
sTfR/ferritin ratio	27.81 [0.22–1070]	21 [1.65–732.70]	32.57 [0.22–1070]	p = 0.0093 at Mann and Whitney test
Serum Hepcidin (ng/mL), Enzyme immunoassay, Peninsula Laboratories, USA; Normal range: [1.71–175.9 ng/mL]	51.14 [0.19–1036]	102.60 [0.76–1036]	30.29 [0.19–437.30]	p< 0.0001 at Mann and Whitney test

MRI: Magnetic Resonance Imaging.

Values are given as median and [range].

### Correlations between liver iron concentration and biological markers of iron metabolism in univariable analysis

**Fig 3:**
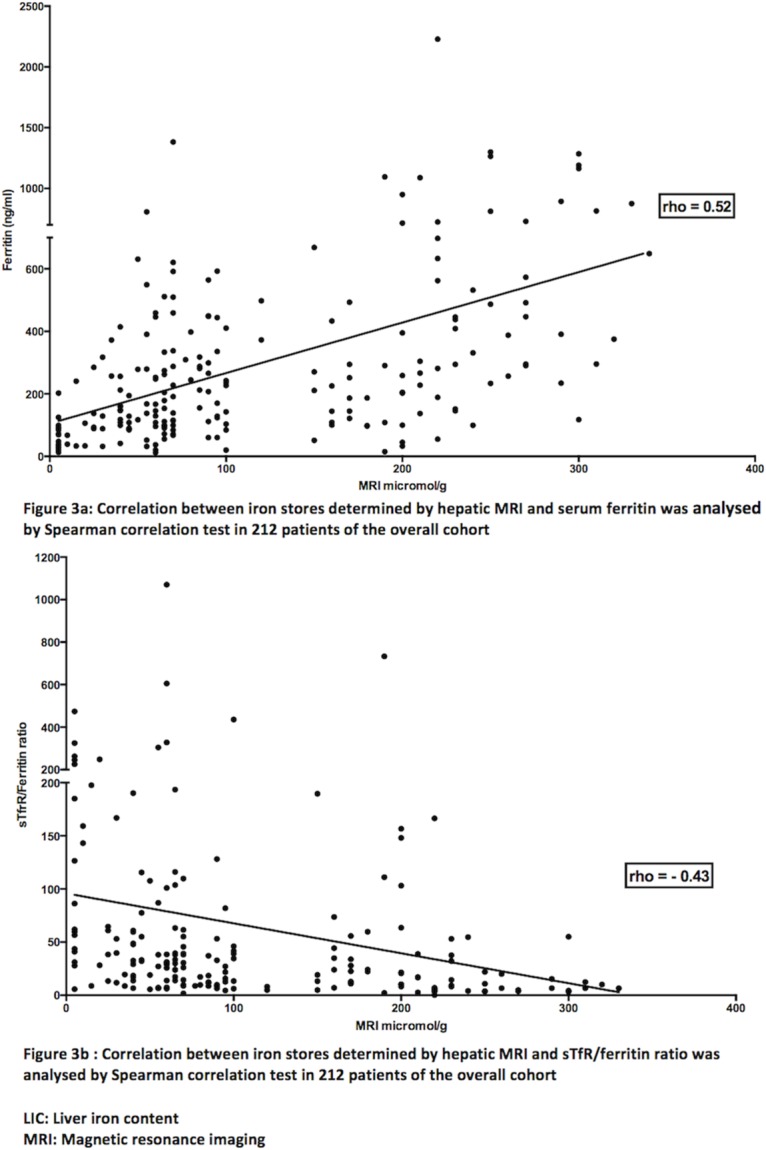
Correlations of liver iron stores studied by quantitative MRI with serum ferritin and the soluble transferrin receptor/ferritin ratio in 212 hemodialysis patients.

Among the biological markers of iron metabolism, only serum ferritin showed a strong correlation (Spearman correlation test) with MRI LIC, with similar results in the whole cohort of 212 patients (rho = 0.52; 95% CI: 0.41–0.61; p< 0.0001), the original cohort of 119 patients (rho = 0.52; 95% CI: 0.37–0.65; p< 0.0001) and the second cohort of 93 patients (rho = 0.47; 95% CI: 0.29–0.62; p< 0.0001) (Table 3 and Fig 3). Weaker but significant correlations were found between LIC and serum iron, serum transferrin, TSAT and hepcidin-25 (p< 0.05, Spearman correlation test), with similar values in the whole cohort and in the two individual cohorts, whereas erythrocyte mean corpuscular volume did not correlate with LIC in any cohort (p> 0.05, Spearman correlation test) (Table 3). Interestingly, the sTfR/ferritin ratio, which is marginally influenced by inflammation [16], also correlated with LIC in the whole cohort and in the two individual cohorts, closer to serum ferritin than those seen with the other iron biomarkers (Table 3 and Fig 3).

**Table 3:** Correlations between the liver iron concentration determined by MRI and iron biomarkers in 212 hemodialysis patients studied (Spearman rank order correlation test).

	Overall Cohort (n°1+n°2)(212 patients)		Cohort n°1 of 119 patients		Cohort n°2 of 93 patients	
Parameter	Spearman rho [95% confidence interval]	P values at test	Spearman rho [95% confidence interval]	P values at test	Spearman rho [95% confidence interval]	P values at test
Serum Ferritin (microg/L)	0.52 [0.41 to 0.61]	p< 0.0001	0.52 [0.37 to 0.65]	p< 0.0001	0.47 [0.29 to 0.62]	p< 0.0001
serum iron (micromol/L)	0.22 [0.09 to 0.35]	p = 0.0011	0.26 [0.07 to 0.42]	p = 0.0048	0.26 [0.05 to 0.44]	p = 0.0128
Serum Transferrin (g/L)	- 0.34 [-0.46 to -0.21]	p< 0.0001	-0.24 [-0.41 to -0.05]	p = 0.0131	-0.35 [-0.52 to -0.15]	p = 0.0006
Serum solubleTransferrin receptors (sTfR) (mg/L)	- 0.12 [-0.27 to 0.02]	p = 0.0868	-0.07 [-0.27 to 0.14]	p = 0.5249	-0.11 [-0.31 to 0.11]	p = 0.3128
TSAT (%)	0.36 [0.24 to 0.48]	p< 0.0001	0.37 [0.20 to 0.52]	p< 0.0001	0.36 [0.16 to 0.53]	p = 0.0004
sTfR/Ferritin ratio	- 0.43 [-0.54 to -0.30]	p< 0.0001	-0.44 [-0.59 to -0.26]	p< 0.0001	-0.36 [-0.53 to −0.16]	p = 0.0004
Hepcidin (ng/mL)	0.42 [0.29 to 0.54]	p< 0.0001	0.34 [0.14 to 0.51]	p = 0.0008	0.42 [0.23 to 0.58]	p< 0.0001
Erythrocyte mean corpuscular volume (fL)	0.04 [-0.10 to 0.18]	p = 0.5637	0.002 [-0.18 to 0.19]	p = 0.99	0.14 [-0.07 to 0.34]	p = 0.1774

Data are given as area of the Spearman rho with the [95% confidence interval].

### Multiple logistic regression analysis

In binary logistic regression analysis, only serum ferritin correctly classified the whole cohort into patients with normal liver iron status (LIC ≤ 50 μmol/g) and those with iron overload (LIC > 50 μmol/g).

In the model including outliers (n = 3), the odds ratio (OR) of ferritin was 1.007 (95% CI: 1.004–1.010) with a Nagelkerke R2 of 0.232 and a classification ability of 75.5%. Conversely, in the model excluding the outliers (n = 3), the odds ratio (OR) of ferritin was 1.009 (95% CI: 1.006–1.013), with a better Nagelkerke R2 (0.308) and a slight improvement in classification ability (76.6%).

### ROC curve analysis of biological markers of iron metabolism for diagnosis of liver iron overload

**Fig 4:**
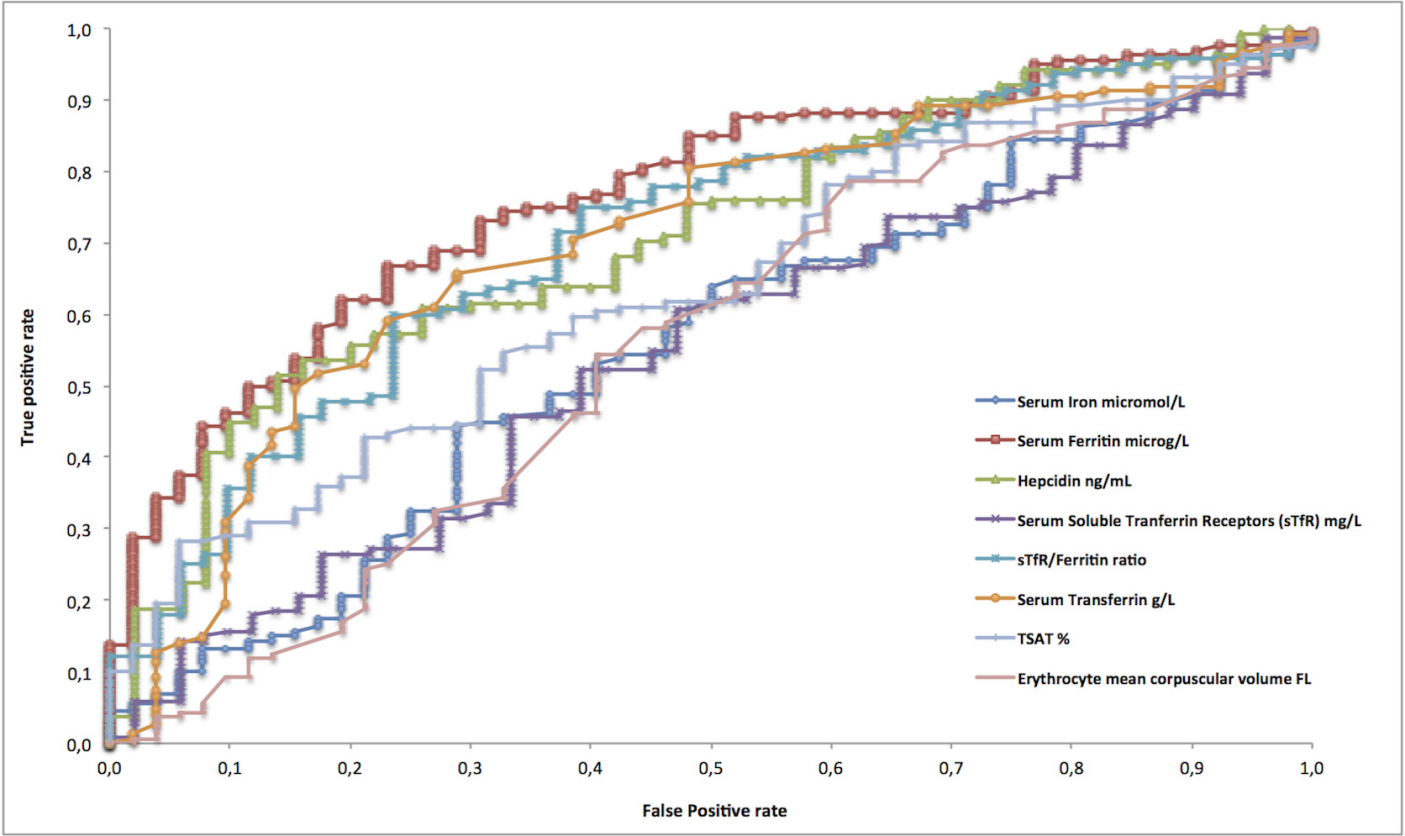
ROC curves of iron biomarkers for predicting iron overload (LIC > 50 μmol/g) in 212 hemodialysis patients studied by quantitative hepatic MRI.

**Fig 5:**
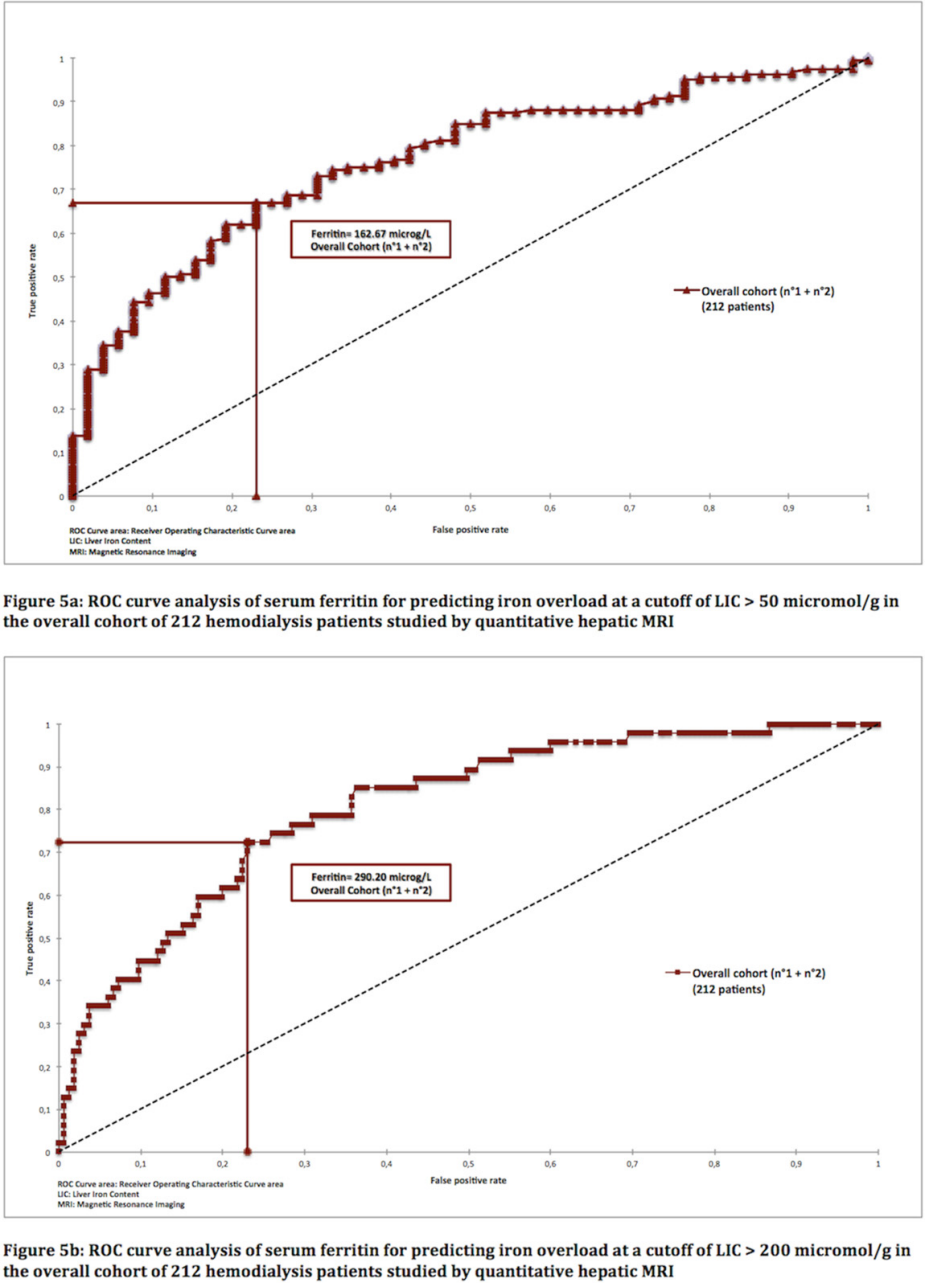
ROC curve analysis of serum ferritin for predicting iron overload at a cutoff of LIC > 50 and > 200 μmol/g in the overall cohort of 212 hemodialysis patients studied by quantitative hepatic MRI.

Serum ferritin was the iron biomarker with the best discriminatory capacity, with an area under the curve (AUC) of 0.767 (95% CI: 0.698–0.835) in the whole cohort of 212 patients (Table 4 and Fig 4). Ferritin also had better diagnostic accuracy than the other iron biomarkers, again with the exception of the sTfR/ferritin ratio (Table 4). The optimal threshold value of serum ferritin for the diagnosis of abnormal hepatic iron stores (LIC > 50 μmol/g) was 162.67 μg/L in the whole cohort; thus, a threshold of 160 μg/L would be convenient in practice (Table 5 and Fig 5). We also generated additional ROC curves for ferritin and the sTfR/ferritin ratio for the prediction of severe iron overload (> 200 μmol/g), a situation at high theoretical risk of clinical complications [9]. The optimal threshold value for serum ferritin for the prediction of severe iron overload (> 200 μmol/g) was 290.20 μg/L (Table 6 and Fig 5).

**Table 4:** Area under the receiver operating characteristics (ROC) curve of iron biomarkers for diagnosis of iron overload (LIC > 50 micromol/g) in 212 hemodialysis patients studied by hepatic MRI.

	Overall Cohort (n°1 + n°2)(n = 212)
Serum iron (micromol/L)	0.556 [0.466 to 0.645] p = 0.223
Serum Transferrin (g/L)	0.703 [0.623 to 0.783] p< 0.0001
Serum Ferritin (microg/L)	0.767 [0.698 to 0.835] p< 0.0001
TSAT (%)	0.634 [0.552 to 0.715] p = 0.001
Serum soluble transferrin receptor (sTfR) (mg/L)	0.545 [0.455 to 0.636] p = 0.327
sTfR/Ferritin ratio	0.709 [0.629 to 0.789] p< 0.0001
Serum Hepcidin (ng/mL)	0.710 [0.631 to 0.789] p< 0.0001
Erythrocyte mean corposcular Volume (fL)	0.556 [0.464 to 0.648] p = 0.233

Data are given as area of the ROC curve with the [95% confidence interval].

**Table 5:** Optimal threshold values and diagnostic accuracy of iron biomarkers to detect iron overload (LIC > 50 micromol/g), as determined in 212 hemodialysis patients studied by hepatic MRI.

	Overall cohort (n°1 + n°2)(n = 212 patients)				
Biochemical marker	Optimal threshold value	Sensitivity	Specificity	Diagnostic accuracy	Likelihood Ratio for a positive test
Serum iron (micromol/L)	10.93	44.40 [36.90–52.10]	71.20 [57.60–81.70]	50.90%	1.54
Serum Transferrin (g/L)	1.90	65.80 [57.80–72.90]	71.20 [57.60–81.70]	67.20%	2.28
Serum Ferritin (microg/L)	162.67	66.90 [59.20–73.70]	76.90 [63.70–86.30]	69.30%	2.90
TSAT (%)	30.27	28.30 [21.90–35.80]	94.20 [83.60–98.50]	44.50%	4.91
Serum solubleTransferrin receptor (sTfR)(mg/L)	5.27	60.70 [52.40–68.40]	52.90 [39.50–65.90]	58.60%	1.29
sTfr/Ferritin ratio	27.84	60 [51.70–67.70]	76.50 [63–86.10]	64.40%	2.55
Serum Hepcidin (ng/mL)	66.81	53.60 [45.30–61.70]	84 [71.10–91.80]	61.70%	3.35
Erythrocyte mean corpuscular volume (fL)	89.75	78.80 [71.70–84.40]	38.50 [26.50–52.10]	68.90%	1.28

Values of sensitivity and specificity are given with [95% confidence interval].

**Table 6:** Area under the receiver operating characteristics (ROC) curve, optimal threshold values and diagnostic accuracy of serum ferritin and the serum soluble transferrin receptor/ferritin ratio to detect severe iron overload (LIC > 200 micromol/g) as determined in 212 hemodialysis patients studied by hepatic MRI.

	Overall cohort (n = 212 patients)					
	ROC Curve Area	Optimal threshold value	Sensitivity	Specificity	Diagnostic accuracy	Likelihood Ratio for a positive test
Serum Ferritin (microg/L)	0.81 [0.74 to 0.87] p< 0.0001	290.20	72.30 [58.10–83.10]	77 [69.90–82.70]	75.9%	3.14
sTfR/Ferritin ratio	0.78 [0.70 to 0.87] p< 0.0001	17.21	75 [58.70–86.30]	71 [63.30–77.50]	71.7%	2.58

Values of area of the ROC Curve are given with the [95% confidence interval].

Values of sensitivity and specificity are given with [95% confidence interval].

**Fig 6:**
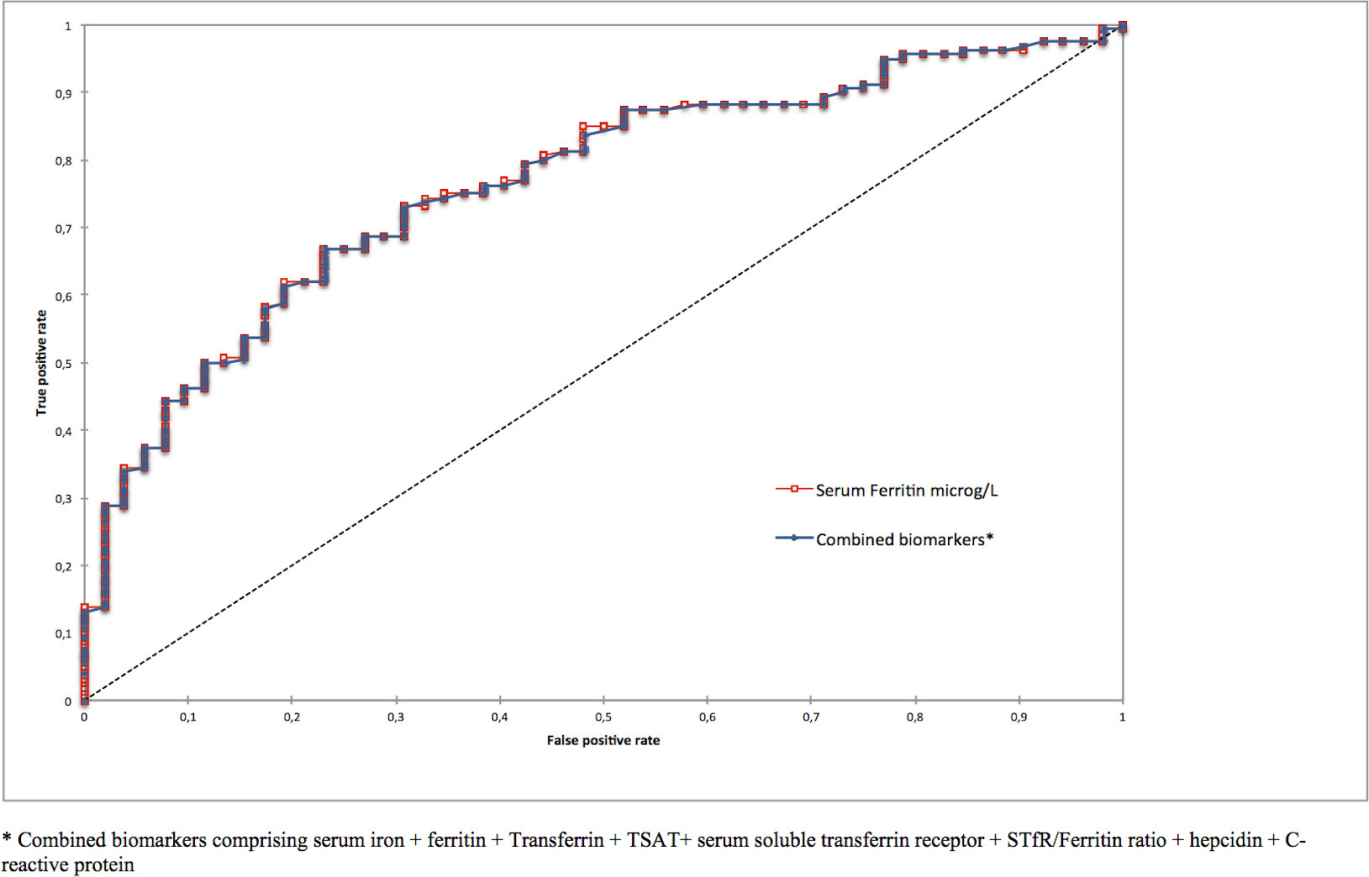
ROC Curves of ferritin compared to a combination of the eight biomarkers for predicting iron overload (LIC > 50 μmol/g) in 212 hemodialysis patients studied by quantitative hepatic MRI.

When ROC curve analysis of the whole cohort excluded the 3 outliers identified by binary logistic regression, the AUC of ferritin rose to 0.798 (95% CI: 0.737–0.859). Interestingly, the optimal threshold of ferritin for the diagnosis of anormal hepatic iron stores (LIC > 50 micromol/g) remained at 162.67 μg/L. The combination of the eight biomarkers (serum iron + ferritin + Transferrin + TSAT+ serum soluble transferrin receptor + STfR/ferritin ratio + hepcidin + C-reactive protein) added no discriminatory diagnostic power for iron overload on MRI (AUC: 0.766; 95%CI: 0.697–0.835) by comparison with ferritin alone (AUC: 0.767; 95% CI: 0.698–0.835) (Fig 6).

## Discussion

To our knowledge this is the fifth study to deal with measurement of hepatic iron stores in dialysis patients by means of quantitative imaging techniques: in the 1990s, Cecchin et al used quantitative computed tomography [21] while, more recently, Canavese et al [22] used the SQUID technique to study LIC in 40 Italian patients; Ferrari et al [23] measured hepatic iron content by magnetic resonance R2 relaxometry in 15 Australian patients with serum ferritin levels well above 500 μg/L; and Ghoti et al [24] recently analyzed LIC by T2*MRI, along with splenic, pancreatic and cardiac iron deposits, in 21 iron-overloaded hemodialysis patients with serum ferritin levels above 1000 μg/L. It should be noted that hepatic iron stores measured by MRI and SQUID are a surrogate marker with as yet unproven clinical relevance in dialysis patients, in terms of mortality and morbidity.

At the end of the 1970s, before the introduction of erythropoietin therapy, several studies demonstrated a correlation between serum ferritin and bone marrow iron content in both hemodialysis patients and non-dialyzed chronic kidney disease (CKD) patients, and concluded that ferritin was a reliable indicator of iron storage levels [25–28]. Regular monitoring of serum ferritin was therefore advocated to guide iron supplementation in CKD patients, particularly those on regular hemodialysis [25,28]. These studies, based solely on bone marrow analysis, which did not take into account Ali’s paradox (low bone marrow iron content despite severe hepatosplenic siderosis) [3], also suggested that the threshold for iron deficiency might be markedly higher in dialysis patients than in normal individuals: various cutoffs, such as 32 μg/L [26], 42 μg/L [25], 50 μg/L [27], and even 82 μg/L [28], were proposed at that time.

With the advent of erythropoietin replacement therapy, ferritin was shown to remain a reliable indicator of iron status, allowing patients at risk of true iron deficiency to be treated prophylactically, mainly (as advocated at that time) with oral iron supplements when the serum ferritin was less than 50 μg/L [29, 30]. More recently, Kalantar-Zadeh et al, studying bone marrow iron stores in 25 anemic CKD patients, 20 of whom were on dialysis, demonstrated that combined determination of ferritin and TSAT was helpful for monitoring iron stores during ESA therapy, especially in case of functional iron deficiency, although only ferritin correlated with bone marrow iron stores, whereas Fernandez-Rodriguez et al showed serum ferritin to be a valuable tool for the diagnosis of true iron deficiency in new dialysis patients awaiting initiation of ESA therapy and studied by bone marrow biopsy [31, 32].

Parenteral iron therapy has gained popularity in the nephrology community in the last fifteen years because of its convenience (infusion during dialysis sessions), its superiority over oral preparations for treating true iron deficiency, and its ability to overcome functional iron deficiency, a very common clinical situation in hemodialysis patients; in addition, this treatment enabled cost savings of about 20%-30% by sparing expensive ESA molecules [1, 2, 8].

Based on bone marrow studies and the lack of known long-term adverse effects, recent guidelines have redefined iron deficiency and iron-store repletion criteria (the KDIGO 2012 target for “upper normal” ferritin in hemodialysis patients is 500 μg/L) and stressed the risk of functional iron deficiency during ESA treatment, leading to greater use of IV iron [1,2,8].

Here, we found that serum ferritin correlated with MRI-determined LIC in a cohort of 212 fit hemodialysis patients without overt inflammation or malnutrition. The relationship between serum ferritin and LIC has been shown to be strongly dependent on the underlying iron-overload disease: the correlation between LIC and serum ferritin in hemodialysis patients seems to be intermediate between genetic hemochromatosis and thalassemia major, where Pearson correlation coefficients (R) as high as 0.72 to 0.80 have been found, and sickle cell disease, where a weaker relationship is reported (R = 0.35) [9, 33, 34].

In our fit hemodialysis patients on ESA and iron maintenance therapy, a serum ferritin level of 160 μg/L was indicative of mild liver iron overload (LIC > 50 μmol/g), whereas a serum ferritin value above 290 μg/L was highly suggestive of severe iron overload (LIC > 200 μmol/g), a situation potentially at a high risk of clinical complications [4,7,8,9]. This ferritin cutoff of 160 μg/L for liver iron store repletion is in very close keeping with results from Mirahmadi et al, who measured bone marrow iron in hemodialysis patients in the late 1970s and found that serum ferritin levels > 124 μg/L were associated with increased bone marrow iron load in this population [26], and also with data from Herbert et al, who showed in the general population that a serum ferritin level > 150 μg/L, when accompanied by a ferritin iron level > 35 ng/ml, was suggestive of iron overload requiring therapeutic intervention [35]; moreover, these latter authors stated that an upper "normal" limit for serum ferritin of 400 μg/L in the general population would be inappropriate, because it would consider as "normal" the 10%-12% of Caucasian Americans and Europeans with heterozygous HFE C282Y hemochromatosis [9,35]. Interestingly, the ferritin cutoff of 290 μg/L found here for severe iron overload is close to the 350 μg/L determined by Bell et al on bone marrow smears in the early 1980s [28], and the 340 μg/L determined by Canavese et al [22] using SQUID in smaller cohorts of patients. Altogether, these results strongly suggest that lower target ranges of ferritin should be considered for hemodialysis patients to avoid the risk of iron overload and improve the safety of iron products [36–38]. The main limitations of this study relate to its observational and cross-sectional design and the exclusion of dialysis patients with overt inflammation and malnutrition, limiting its generalization. Thus, a prospective interventional study is required to confirm the ability of these new upper ferritin targets to protect hemodialysis patients from iron overload. Finally, there is also a need for a lengthy, prospective, randomized trial comparing low, medium and high targets for “upper normal” ferritin levels in hemodialysis patients, based on serial MRI (twice yearly) and an analysis of morbidity and mortality to endorse the findings reported here and in recent epidemiological studies [10–13].

## Conclusion

Among the biological markers of iron metabolism examined here, serum ferritin correctly reflected liver iron stores as assessed by MRI in hemodialysis patients. Moreover, serum ferritin accurately predicted the degree of hepatic iron overload. Finally, ferritin target values for hemodialysis patients should be lowered to avoid the risk of iron overload.

## Supporting Information

S1 Text TREND Statement Checklist. (PDF)

S2 Text Trial Study protocol (in French) submitted to and approved by the COMEDIMS before the trial began. (DOC)

S3 Text Trial Study Protocol (translated in English) submitted to and approved by the COMEDIMS before the trial began. (DOC)
