# Supplementary material for: Reassessment of Iron Biomarkers for Prediction of Dialysis Iron Overload: An MRI Study
Source: PLoS One. 2015 Jul 16;10(7):e0132006. doi: 10.1371/journal.pone.0132006 (PMC4504469; doi:10.1371/journal.pone.0132006)
Supplement: S2 Text — (DOC) [file pone.0132006.s002.doc]

**S2**_Trial Study protocol (in french) submitted to and approved by the COMEDIMS before the trial began.

**Enregistrement de l’étude dans un registre d’essais cliniques :** Sera réalisé s’il est requis par le COMEDIMS

**Approbation du Comité:** Protocole soumis au COMEDIMS (Commission du Médicament, des dispositifs Médicaux Stériles) du CHP Claude Galien et approuvé le 09/12/2004.

**TItre:** Une étude de cohorte de patients hémodialysés basée sur l’imagerie par résonance magnétique hépatique.

**Titre Scientifique:** Détermination et Analyse des stocks de fer hépatique de patients hémodialysés par imagerie par résonance magnétique : une étude observationnelle crossectionelle et longitudinale

**Lieu:** Service de Néphrologie et d’Hémodialyse, Hôpital Privé Claude Galien, Générale de Santé, 20 route de Boussy, 91480 Quincy sous Sénart, France

**Dates de l’étude:** Janvier 2005 à Janvier 2015

**Type d’étude**: Etude monocentrique observationnelle, prospective, crosssectionnelle et longitudinale

**But et Hypothèse de l’étude**:

Le but de cette étude est de déterminer le stock de fer hépatique en utilisant l’imagerie par résonance magnétique (IRM) en utilisant l’algorithme de l’Université de Rennes, pour mesurer la quantité de fer dans le foie, et la méthode R2* pour étudier la charge ferrique de la rate et le cœur dans une cohorte de patients hémodialysés, recevant d’une part du fer intraveineux et d’autre part des agents stimulant l’érythropoïèse (Erythropoïétine EPO) en respectant les recommandations actuelles de bonne pratique.

Notre hypothèse est que les stocks de fer pourraient être augmentés par l’utilisation excessive de fer iatrogène comme récemment démontré par la technologie SQUID dans une petite cohorte de patients italiens (Canavese C, Kidney Int 2004; 65(3): 1091-1098). En outre, l’IRM hépatique avec l’algorithme the Rennes a été récemment montré comme corrélé de façon très forte avec le contenu en fer hépatique (liver iron content: LIC) déterminé sur des biopsies hépatiques de patients souffrant de maladies hépatiques, d’hémochromatose génétique et d’hémosidérose secondaires permettant une estimation non invasive et fiable du contenu hépatique en fer (Gandon et al. Lancet 2004 Jan 31; 363(9406): 357-362).

L’étude s’attachera à déterminer aussi les facteurs de risque de surcharge en fer et spécialement le rôle du traitement par fer intraveineux.

**Méthodologie**:

**Etude non randomisée et monocentrique observationnelle chez des patients hémodialysés:**

**1. Détermination de la quantité de fer dans le foie, la rate et le cœur par IRM selon la méthode de Rennes (foie) et R2* (rate et cœur).**

Les IRM pour la quantification des stocks de fer seront réalisées avec un délai d’au moins sept jours après la dernière injection de fer.

**Pourcentage de patients avec un stock de fer hépatique anormal (faible, moyen, modéré, et surcharge sévère)**

- Comme l’IRM hépatique détecte précisément la surcharge de fer dans le foie au-dessus de 50 μmol/g, la limite supérieure à la normale a été fixée à 50 μmol/g pour cette étude

- Les valeurs entre 51 et 100 μmol/g seront considérées représenter une surcharge faible en fer, les valeurs entre 101 et 200 μmol/g: une surcharge modérée; et les valeurs > 200 μmol/g: une surcharge sévère.

**2. Caractéristiques des patients hémodialysés étudiés**: Age, Genre, Temps passé en dialyse, Traitement par érythropoïétine, Traitement parentéral par fer, Index des comorbidités de Liu, Index des comorbidités de Charlson, Pourcentage de patient diabétiques, Score Audit pour la consommation Alcool.

**3. Mesures des marqueurs biologiques du métabolisme du fer (réalisées en routine sauf le dosage d’hepcidine)**: Hémoglobine, Volume globulaire moyen, Protéine C-réactive, Ferritine sérique, Fer sérique, Transferrine, Saturation de la Transferrine (TSAT), Récepteurs solubles de la transferrine (sTfR), Hepcidine-25 sérique.

**Nombre cible de participants**: 400

**Participants – Critères d’inclusion:**

**1.** Patients en insuffisance rénale chronique terminale relevant de l’épuration extra-rénale chronique traités depuis au moins 3 mois, par hémodialyse (en bi-puncture avec dialysat ultrapure en bicarbonate, sans réutilisation des membranes) ou par hémodiafiltration 3 fois par semaine.

**2.** Patients des deux sexes et âgés de plus de 18 ans

**Participants – Critères d’exclusion:**

**1.** Refus de participer à cette étude

**2.** Mauvaise observance des traitements en hémodialyse

**3.** Age < 18 ans

**4.** Pace maker cardiaque, valves métalliques cardiaques, et débris métalliques dans le corps

**5.** Déficience cognitive sévère

**6.** Claustrophobie

**7.** Cirrhose hépatique

**8.** Maladie inflammatoire ou infectieuse

**9.** Malnutrition

**10.** Saignement majeur récent (< 3 mois), chirurgie majeure (< 3 mois), dépendance de transfusion, transfusion récente (< 3 mois)

**11.** Cancer en phase évoluée
