# Supplementary material for: Reassessment of Iron Biomarkers for Prediction of Dialysis Iron Overload: An MRI Study
Source: PLoS One. 2015 Jul 16;10(7):e0132006. doi: 10.1371/journal.pone.0132006 (PMC4504469; doi:10.1371/journal.pone.0132006)
Supplement: S3 Text — (DOC) [file pone.0132006.s003.doc]

**S3**_Trial Study Protocol (translated in english) submitted to and approved by the COMEDIMS before the trial began.

**Trial Registration:** will be performed if required by the COMEDIMS

**Ethics approval:** Presented at COMEDIMS (Drug, Devices and Clinical Trials Committee) CHP Claude Galien and approved on 09/12/2004.

**Tittle:** A cohort study of hemodialysis patients based on hepatic magnetic resonance imaging.

**Scientific Tittle:** Analysis of hepatic iron stores of hemodialysis patients by magnetic resonance imaging: a cross-sectional and longitudinal study

**Location:** Division of Nephrology and Dialysis, Private Hospital Claude Galien, Générale de Santé, 20 route de Boussy, 91480 Quincy sous Sénart, France

**Dates:** January 2005 to January 2015

**Study design**: Prospective cross-sectional and longitudinal monocentric observational study

**Study Hypothesis**:

The aim of this study was to determine hepatic iron content, using magnetic resonance imaging (MRI) with the Rennes University algorithm, to measure the iron content of the liver, and R2* for spleen and heart in a cohort of hemodialysis patients receiving both intravenous iron and erythropoiesis stimulating agents (ESA), in keeping with current guidelines. We hypothesized that iron stores may be increased by excessive iatrogenic iron repletion as recently demonstrated by SQUID in a small cohort of italian patients (Canavese C, Kidney Int 2004; 65(3): 1091-1098). Moreover, hepatic MRI with the Rennes algorithm has been recently shown to correlate closely with liver iron content (LIC) on hepatic biopsies of patients with liver diseases, genetic hemochromatosis and secondary hemosiderosis allowing a non invasive and accurate estimation of LIC (Gandon et al. Lancet 2004 Jan 31; 363(9406): 357-362).

The study will also find out about the risk factors of iron overload, and especially the role of iron

therapy.

**Methodology**:

**Non-randomised and monocentric observationnal study on hemodialysis patients:**

**1.** **Determination of iron content (μmol/g/dry weight) by MRI in liver, spleen and heart**.

MRI for quantification of iron stores will be performed at least seven days after iron infusion.

**Percentage of patients with abnormal iron liver content (mild, moderate and severe overload)**

- As hepatic MRI accurately detects liver iron overload exceeding 50 μmol/g, the upper limit of normal will be set at 50 μmol/g for this study

- Values between 51 and 100 μmol/g will be considered to represent mild iron overload, values between 101 and 200 μmol/g moderate iron overload and values > 200 μmol/g severe iron

overload.

**2. Characteristis and findings** in hemodialysis patients : Age, Gender, Dialysis vintage, ESA Therapy, Parenteral Iron therapy, Liu Comorbidity Index, Charlson Comorbidity Index, Diabetic patients percentage, Audit Alcool Score.

**3. Measurements of routine biochemical markers of iron metabolism (with the exception of hepcidin)**: Hemoglobin, Erythrocyte mean corpuscular volume, C-reactive protein, Serum Ferritin, Serum iron, Transferrin, Transferrin Saturation (TSAT), Soluble transferrin receptors (sTfR), Serum Hepcidin-25.

**Target number of participants**: 400

**Participants – Inclusion criteria:**

**1.** Chronic kidney disease (CKD) patients on hemodialysis for at least 3 months, Undergoing chronic intermittent bipuncture bicarbonate hemodialysis (with ultrapure dialysate single use biocompatible membranes) or hemodiafiltration three times a week

**2.** Patient of either sex and aged 18 years and older

**Participants – Exclusion criteria:**

**1.** Refusal to participate in the study

**2.** Poor compliance with the dialysis therapy schedule

**3.** Age < 18 years

**4.** Cardiac Pace maker, metallic cardiac valves, and metallic debris

**5.** Severe cognitive impairment

**6.** Claustrophobia

**7.** Hepatic cirrhosis

**8.** Overt inflammatory or infectious disease

**9.** Malnutrition

**10.** Recent major bleeding (< 3 months), major surgery (< 3 months), transfusion dependency, recent transfusion (< 3 months)

**11.** Intractable malignancy
